# Childhood abuse and opioid prescription use in adulthood: Differences between non-Hispanic Whites and non-Hispanic Blacks in the United States

**DOI:** 10.1371/journal.pone.0291752

**Published:** 2023-09-21

**Authors:** Hee Yun Lee, Jieun Song, Eun Young Choi

**Affiliations:** 1 School of Social Work, The University of Alabama, Tuscaloosa, AL, United States of America; 2 Institute on Aging, University of Wisconsin-Madison, Madison, WI, United States of America; 3 Leonard Davis School of Gerontology, University of Southern California, Los Angeles, CA, United States of America; University of Wisconsin-Milwaukee Joseph J Zilber School of Public Health, UNITED STATES

## Abstract

Despite the rapid rise in opioid prescription medication usage, little research has examined the role of early life adversity, such as childhood abuse, particularly in the context of race, in opioid prescription usage in adulthood. Guided by the life course perspective, the current study investigates whether experiencing childhood abuse increases the risk of opioid prescription use in adulthood and whether this association varies by race. Data were sourced from the second wave of the Midlife in the United States (MIDUS) study (2004–2005). The analytic sample consisted of two groups: non-Hispanic Whites (*n* = 714) and non-Hispanic Blacks (*n* = 151). Opioid prescription use was identified from the participants’ medication list using the MULTUM Lexicon Drug Database Classification System. Three types of childhood abuse—emotional, physical, and sexual—were assessed via summary scales derived from the Childhood Trauma Questionnaire. The results indicate a significant interaction between childhood physical abuse and race. Among non-Hispanic Whites, higher exposure to physical abuse during childhood was associated with greater odds of opioid prescription use in adulthood, even after adjusting for chronic pain, physical and mental health, and sociodemographic characteristics. However, the association between childhood physical abuse and opioid prescription use in adulthood was non-significant among non-Hispanic Black individuals. These findings underscore the long-term adverse health effects of physical abuse in childhood, particularly for non-Hispanic Whites, and suggest support for developing and implementing tailored intervention strategies.

## Introduction

Over the past few decades, the use of opioid prescription medications has grown rapidly in the United States [1]. In 2018, the proportion of individuals who reported taking at least one opioid prescription in the past year reached 15% (40 million people) [2]. The rapid increase in opioid prescription use has raised concerns about the risk of opioid misuse and addiction. The use of opioid prescription medications is associated with an increased risk of depression and anxiety disorders [3] and opioid misuse [4], overdose [5], heroin use [6], and deaths [7]. Annually, an estimated $78.5 billion is spent in the United States to address opioid prescription overdose, abuse, and addiction [8], reflecting the substantial costs of opioid misuse at personal and societal levels.

In response, prior research has identified multiple predictors of opioid prescription use, including demographic risk factors (e.g., older age, being female, widowed or divorced, and Caucasian [2,9,10], socioeconomic status (e.g., less education and unemployment), and poor physical health [9,11,12]. However, existing literature has mostly focused on concurrent individual-level characteristics, limiting our understanding of the risk associated with life course experiences such as adverse childhood experiences (ACEs). This study is guided by the life course perspective [13], specifically the concept of cumulative advantage/disadvantage [14], which posits a “systemic tendency for inter-individual divergence in a given characteristic (e.g., money or health status),” especially over time [14,15]. Individuals with early disadvantages are more vulnerable to the detrimental impact of adverse circumstances than their counterparts with fewer disadvantages. Those who experienced childhood abuse may be more vulnerable to opioid prescription use throughout adulthood. Studies show a significant link between childhood abuse and the prevalence of pain-related conditions and addiction in adulthood [16,17].

Despite the well-established association of adverse childhood experiences with opioid misuse [18] and overdose [19], little is known about the role of ACEs in using opioid prescription medications, which can be a precursor to or substantial risk factor for opioid abuse and addiction. For example, among adolescents and young adults, those who used opioid prescription medications had a 33% increase in the risk of future opioid misuse [4] and a 5% increase in the risk of the diagnosis of opioid abuse [20]. To our knowledge, only one study has examined the association between childhood abuse and opioid prescription use in adulthood [21]. Using data from the National Longitudinal Study of Adolescent to Adult Health, the authors found that physical and emotional abuse during childhood is linked to opioid prescription use among adults aged 24–32. The authors suggested biological (i.e., lower pain thresholds and chronic pain in adulthood) and psychological (i.e., negative affective states and post-traumatic stress symptoms) pathways explain a higher rate of opioid prescription use among those with a history of childhood abuse. However, there is still no empirical evidence as to whether the influence of childhood abuse on opioid prescription use extends beyond the period of young adulthood. Given that exposure to opioid prescriptions is highest among middle-aged and older adults [2,9], it is essential to examine whether childhood abuse is associated with opioid prescription use with a broad age range sample, including middle-aged and late adulthood.

Furthermore, we aim to examine whether the association between childhood abuse and opioid use in adulthood varies by race. Exposure to childhood abuse places all individuals at an elevated risk of deleterious health and behavioral problems, yet not everyone is equally affected by such a traumatic life event. Race is a key dimension of social stratification through which economic, social, and cultural resources are allocated differently, subsequently affecting individuals’ capacity to cope with adverse experiences. To our knowledge, no studies have specifically investigated potential racial discrepancies in the association between childhood abuse and opioid prescription usage. Nonetheless, several factors suggest the presence of such disparities. Non-Hispanic Black (hereafter Black) individuals are more likely to report adverse childhood experiences than their non-Hispanic White (hereafter White) counterparts [22] and also face a disproportionately heightened risk of psychiatric disturbances, such as PTSD, due to exposure to traumatic events [23]. This disparity suggests they may be more inclined to receive opioid prescriptions for chronic pain due to compromised health, elevating their risk of opioid prescription utilization. Conversely, a compelling body of literature exists on the undertreatment of pain among Black individuals, indicating that they are less prone to opioid prescriptions than Whites with comparable pain conditions or traumatic experiences. For instance, Blacks are significantly less likely to receive opioid prescriptions for pain management, at a rate of 29% less than Whites [24], particularly for severe pain [25]. This disparity has been ascribed to misperceptions among medical providers regarding: a heightened risk of dependency or misuse, lower accuracy in pain reporting, or a greater pain tolerance among Blacks compared to Whites [26,27].

In sum, despite the surging importance of opioid prescription use as a significant precursor of the opioid epidemic and its substantial consequences at public health levels, there is a dearth of research on childhood abuse as a possible predictor of opioid prescription use in adulthood and its potential variance across racial groups. The current study addresses this gap by analyzing data from a national sample of adults aged 35–84 and examines whether experiencing three types of childhood abuse (emotional, physical, and sexual abuse) is associated with opioid prescription use in adulthood, contingent on race. Our findings will provide information that can be utilized in developing tailored interventions for those at greater risk of opioid prescription use.

## Materials and methods

### Data and sample

We analyzed data from the Midlife in the United States (MIDUS) study, a longitudinal survey of a national probability sample of non-institutionalized, English-speaking adults aged 25 to 74 during the initial study in 1995–1996 (MIDUS 1). Respondents were surveyed again in 2004–2006 when they were aged 35 to 84 (MIDUS 2) and in 2013–2014 (MIDUS 3) [28,29].

The current study analyzes data from MIDUS 2 participants who completed the survey and biomarker data collection. In addition to the survey (interview and questionnaire data), biomarker data was collected from a sub-sample of MIDUS participants at the General Clinical Research Centers (GCRC) at the University of Wisconsin, University of California Los Angeles (UCLA), and Georgetown University. The participants brought all their current medications in the original bottles to their GCRC visit. During their visit, the study staff recorded the medication name, dosage, and route of administration (how often the medication is taken, how long the participant has been taking it, and why the participant thinks s/he is taking it). Prescription medication includes all FDA-approved medications prescribed by an authorized or licensed medical provider under the Western medical tradition [30].

In the next step, medication names were linked to Generic Names and corresponding DrugIDs via linkage to the Lexi-Data database and then linked to their associated therapeutic and pharmacologic class codes (NOTE: The Lexicomp® Lexi-Data base is a proprietary database. According to the license with Lexicomp®, users of data derived from the Lexi-Data file should include the following in any publications or presentations: “Lexi-Data is a drug data solution offered by Wolters Kluwer Clinical Drug Information. Certain information about therapeutic effects and active ingredients of generic medications identified in the MIDUS (Midlife in the U.S.) study is derived from the Lexi-Data database and used under license from Lexi-Comp, Inc., which reserves all rights in that information.” (See Documentation for MIDUS Medication Data for details) [31]. Participants also completed a medical history and a self-administered questionnaire that included childhood abuse items.

Of the 5,555 MIDUS 2 survey participants, 1,255 agreed to participate and completed biomarker data collection, and 865 of them were identified as taking at least one prescription medication with further information of medication for pharmacologic class (either opioid or others) and were included in the analysis. Non-prescription medication users (n = 258) significantly differed from the two prescription medication users in age and gender, which would raise concerns about the confounding effects of age and gender in examining the correlates of opioid prescription medication use. As a result, the two prescription user groups (86 opioid vs. 779 non-opioid) were analyzed in the current study. The time between the survey and biomarker data collection was 24 months on average. MIDUS data collection was reviewed and approved by the Education and Social/Behavioral Sciences and the Health Sciences IRBs at the University of Wisconsin-Madison. Informed written consent was obtained for all participants.

### Measures

*Opioid prescription use*. Opioid prescription usage status was identified based on the participants’ medication chart that research staff or nurses filled during the participants’ overnight visit to GCRC during MIDUS 2 Biomarker study. The participants were asked to bring all the medications (prescription, OTC, alternative) they took at the time of the MIDUS 2 Biomarker study. Multum Pharmacologic Class defined opioid prescription as “if a medication was classified into Multum Pharmacological Category 946: Analgesic/opioid, the participant was identified as an opioid prescription user.” Subsequently, participants taking prescription medications other than opioids were identified as “non-opioid prescription medication users.” Finally, those who did not take prescription medications were identified as “non-prescription medication users” and excluded from the analytic sample.

The most commonly used opioid prescription within the analytic sample (49.5%) was Hydrocodone and followed by Tramadol (15.1%), Fentanyl (8.6%), Codeine (7.5%), Morphine (6.5%), Oxycodone (5.4%), and Methadone (3.2%) (note that the sum exceeds 100% because some patients reported two opioid prescription medications).

*Race and ethnicity*. Participants self-reported their racial origin (White, Black or African American, Native American or Alaska Native/Eskimo, Asian, Native Hawaiian or Pacific Islanders, other) and ethnicity (non-Hispanic, Hispanic). Due to the small number of participants from specific racial and ethnic groups other than non-Hispanic Whites or Black, we included only non-Hispanic White and non-Hispanic Black individuals in the analytic sample (1 = Whites, 0 = Blacks). Of the 865 individuals who participated in MIDUS 2 biomarker data collection and were identified as prescription medication users, 86 were using opioid prescription medication (60 Whites and 26 Blacks), and 779 were using non-opioid prescription medication (654 Whites and 125 Blacks).

*Childhood abuse*: *emotional*, *physical*, *and sexual abuse*. Childhood abuse experience was assessed via items in the Childhood Trauma Questions (CTQ) scale [32], specifically with the items for emotional, physical, and sexual abuse analyzed in the current study. Each abuse scale consists of five specific items asking about abuse experiences during childhood, such as “people in my family said hurtful or insulting things to me” (emotional abuse), “people in my family hit me so hard that it left me with bruises or marks” (physical abuse), and “someone tried to touch me in a sexual way, or tried to make me touch them” (sexual abuse). A complete list of the items is available in Supplemental Documents. Participants’ responses to these items were coded as follows: 0 = never true, 1 = rarely true or sometimes true, and 2 = often true or very often true. The final score for each abuse form was then calculated as the average of the responses to its corresponding five items, resulting in a score ranging between 0 and 2 for each scale.

*Covariates*. The analyses included several control variables that past research has shown to be significantly associated with opioid use: age, sex, education, marital status, and physical and mental health status [33,34]. *Poor/fair physical health* was measured by the participant’s self-assessment: “In general, would you say your physical health is excellent, very good, good, fair, or poor?” The response was recoded into 1 (fair or poor) or 0 (excellent, very good, or good) to indicate whether the participant’s subjective physical health was poor/fair or not. *Chronic Pain beyond normal* was assessed by asking, “Do you have chronic pain, that is, do you have pain that persists beyond the time of normal healing and has lasted anywhere from a few months to many years?” (1 = yes, 0 = no). Those who answered affirmatively were coded as having chronic pain beyond normal, and the others were coded as not having chronic pain (1 or 0). *Depressive symptoms* were assessed by self-report for the items in the Center for Epidemiologic Studies Depression Scale (CES-D) in the questionnaire during the biomarker data collection [35]. The score ranged from 0 to 54, with greater scores indicating greater levels of depression. A continuous value was included in the analysis. *Education* was assessed by the participants’ self-reported highest education completed (0 to 20 years, top-coded at 20). *Marital status* was assessed by the participant’s report of the current marital status at the interview (1 = currently married, 0 = unmarried [separated, divorced, widowed, never been married]).

### Analysis plan

Descriptive characteristics of the four study groups (White opioid prescription users, White non-opioid prescription users, Black opioid prescription users, and Black non-opioid prescription users) were compared using a one-way analysis of variance, with Duncan post-hoc tests for between-group contrasts. Subsequently, logistic regression evaluated childhood abuse and race as predictors of opioid prescription use in adulthood. We examined differences in the association between different types of childhood abuse experience (emotional, physical, and sexual abuse) and opioid prescription use in adulthood, contingent on race, using childhood abuse experience × race interaction term in three separate regression models, controlling for sociodemographic factors and physical and mental health status that might have affected opioid prescription use. The missingness of analytic variables was between 0 to 1.6%. Complete case deletion was applied for the small number of cases with missing data. For the childhood abuse scales, each scale consists of five items, and scale scores were computed by summing all items for the cases without missing data. In the case of only one missing value, mean substitution was used to calculate the score of each scale, and cases with more than one missing value were treated as missing on the scale score.

## Results

Table 1 presents descriptive statistics for participants with an opioid or a non-opioid prescription, further divided by race (Whites vs. Blacks). Significant differences were found among the groups in all sociodemographic characteristics, mental and physical health, and childhood abuse experiences. White participants with non-opioid prescriptions (Group B) had higher levels of educational attainment (15 years) than White participants with opioid prescriptions (Group A: 13.9 years) and all Black participants, regardless of opioid prescription status (Groups C: 13.5 years and D: 13.3 years). Further, the Black participants with opioid prescriptions (Group C) were more likely to report poor/fair physical health (73.1%) than other participants (Groups A: 30%, B: 7.8%, and D: 34.4%). Regardless of race, participants with opioid prescriptions (Groups A and C) were more likely to experience chronic pain as defined as pain persisting beyond the time of normal healing (A: 75.9% and C: 69.2%) and reported higher exposure to sexual abuse in childhood (A: 0.50 and C: 0.58) than those with non-opioid prescriptions (B: 0.26 and D: 0.30). Overall, 8% (60 out of 714) of White prescription users had at least one opioid prescription, while among Black prescription users, 17% (26 out of 151) had received at least one opioid prescription.

**Table 1:** Descriptive analysis of opioid prescription medication users vs. Non-opioid prescription medication Users in Whites and Blacks (*N* = 865).

	Whites		Blacks		Group Comparison ^b^
	Opioid Rx Users (A)	Non-opioid Rx Users (B)	Opioid Rx Users (C)	Non-opioid Rx Users (D)	
	M (SD)	M (SD)	M (SD)	M (SD)	
Age	58.1 (11.2)	60.4 (11.6)	56.1 (10.3)	56.7 (10.5)	**
Female, %	58.3	57.5	65.4	71.2	*
Education (years)	13.9 (2.3)	15.0 (2.5)	13.5 (3.1)	13.3 (2.4)	*** B > A,C,D
Married, %	70.0	76.3	42.3	28.0	*** A,B > C,D
**Current Health**					
Poor/fair health, % ^a^	30.0	7.8	73.1	34.4	*** C > A,D > B
Chronic pains beyond normal, %	75.9	32.9	69.2	29.6	*** A,C > B,D
Depressive symptoms	13.1 (10.6)	7.7 (7.4)	16.2 (11.6)	11.4 (8.9)	*** A > C,D > B
**Childhood Abuse**					
Emotional abuse	.92 (.67)	.66 (.58)	.77 (.71)	.63 (.63)	* A> B,C,D
Physical abuse	.77 (.62)	.55 (.53)	.77 (.51)	.77 (.54)	*** A,C,D > B
Sexual abuse	.50 (.70)	.26 (.53)	.58 (.86)	.30 (.60)	*** A,C > B,D
N	60	654	26	125	

*Note*. M = Mean; SD = standard deviation.

^a^ 1 = poor/fair health, 0 = good/very good/excellent health.

^b^ One-way ANOVA and Duncan post-hoc tests were conducted for the group comparisons.

* *p* < .05, ** *p* < .01, *** *p* < .001.

The base model, which included demographic characteristics, mental and physical health, showed that significant predictors of opioid prescription use were physical health, chronic pain, and depressive symptoms. Participants who reported poor or fair physical health (Exp(B) = 2.819 [CI = 1.546, 5,140], *p* = .001), chronic pain beyond normal (Exp(B) = 4.469 [CI = 2.615,7.636], *p* = .000), and higher levels of depressive symptoms (Exp(B) = 1.030 [CI = 1.003, 1.058], *p* = .027) were more likely to receive an opioid prescription.

Table 2 presents the results of logistic regression models predicting opioid prescription use in adulthood, with emotional/physical/sexual abuse experienced in childhood and physical and mental health and sociodemographic characteristics in adulthood.

**Table 2:** Logistic regression models predicting opioid prescription medication use: Effects of childhood abuse in Whites and Blacks.

	Opioid Prescription Medication Use ^a^					
	OR [95% CI]	*p*	OR [95% CI]	*p*	OR [95% CI]	*p*
Age	0.987 [0.964–1.010]	0.252	0.988 [0.966–1.011]	0.306	0.988 [0.966–1.011]	0.292
Female vs. Male (ref.)	0.954 [0.561–1.623]	0.863	0.981 [0.576–1.670]	0.943	0.877 [0.509–1.511]	0.637
Whites vs. Blacks (ref.)	0.426 [0.170–1.067]	0.069	0.245 [0.091–0.662]	0.006	0.572 [0.269–1.218]	0.147
Education (years)	0.918 [0.828–1.018]	0.104	0.924 [0.834–1.025]	0.134	0.923 [0.832–1.023]	0.126
Married vs. unmarried (ref.)	1.246 [0.690–2.250]	0.466	1.279 [0.707–2.314]	0.416	1.370 [0.757–2.480]	0.298
Self-rated health: poor/fair vs. good/very good/excellent (ref.)	2.754 [1.513–5.010]	<0.001	2.921 [1.605–5.316]	<0.001	2.802 [1.532–5.124]	<0.001
Chronic pains beyond normal vs. no chronic pain (ref.)	4.517 [2.637–7.737]	<0.001	4.749 [2.755–8.186]	<0.001	4.397 [2.564–7.542]	<0.001
Depressive symptoms	1.032 [1.004–1.061]	0.025	1.031 [1.004–1.059]	0.022	1.031 [1.003–1.059]	0.027
Emotional abuse in childhood	0.694 [0.335–1.439]	0.326	--	--	--	--
Emotional abuse in childhood × Race	0.694 [0.335–1.439]	0.326	--	--	--	--
Physical abuse in childhood	--	--	0.446 [0.194–1.023]	0.057	--	--
Physical abuse in childhood × Race	--	--	3.996[1.499–10.655]	0.006	--	--
Sexual abuse in childhood	--	--	--	--	1.121 [0.597–2.106]	0.721
Sexual abuse in childhood × Race	--	--	--	--	1.493 [0.702–3.176]	0.297
Overall Model Statistics						
*Log Likelihood χ* ^*2*^	97.68	<0.001	102.36	<0.001	100.48	<0.001

Note: OR (odds ratio) was presented with a 95% Confidence Interval. CI = Confidence interval.

^a^ Reference *=* non-opioid prescription medication use.

**Fig 1:**
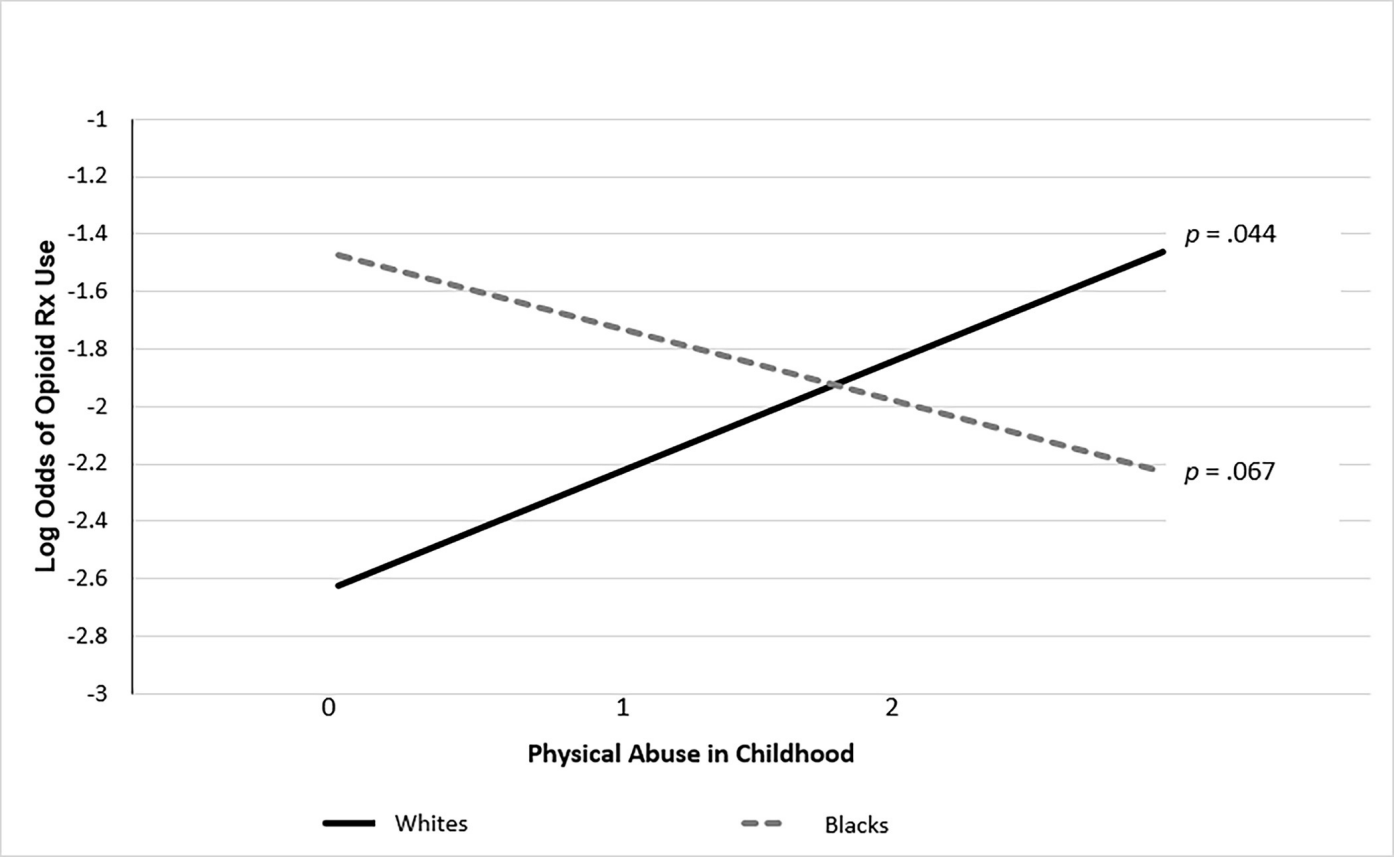
Predicted log odds of opioid prescription medication use contingent on physical abuse in childhood and race. *Note*. The values represent the average responses to the 5 items on physical abuse; 0 = never true, 1 = rarely true or sometimes true, and 2 = often true or very often true.

Table 2 also presents the findings from a logistic regression analysis that examined the potential moderating effects of race on the association between childhood abuse experiences and opioid prescription use in adulthood. The results showed a significant interaction effect between childhood physical abuse and race (OR = 3.996, 95% CI [1.499–10.655], *p* = .006)). Fig 1 illustrates that, among the White participants, greater exposure to physical abuse during childhood was associated with greater log odds of opioid prescription use in adulthood (b = .546, *p* = .044). On the other hand, the association between childhood physical abuse and opioid prescription use in adulthood was insignificant among Black participants (b = -.878, *p* = .067).

## Discussion

The current study showed significant racial differences in the effects of childhood abuse on opioid prescription use compared to non-opioid prescription use in adulthood. Specifically, as shown in Table 2, the adverse effects of experiencing physical abuse in childhood on opioid use in adulthood were more pronounced in White adults than in Black adults (OR = 3.996, *p* = .006). Notably, the current findings provide the first evidence of group differences among those who experienced childhood abuse and are opioid prescription medication users and call for further investigation of the mechanisms underlying the racial differences.

Among Whites, experiencing physical abuse in childhood significantly increased the probability of opioid prescription use in adulthood, even after adjusting for sociodemographic characteristics (age, gender, education, marital status) and health status (physical health, depressive symptoms, chronic pains). These results align with prior research adopting the life course perspective on opioid prescription use. For example, a study by Austin et al. reported that childhood emotional and physical abuse, but not sexual, was associated with the recent use of opioid prescription medications during early adulthood [21]. Different results can be attributable to differences in the measures of abuse assessment and the sample’s age range (binary abuse measure in younger adults in the Austin and colleagues’ study vs. ordinal abuse measure and full age range of adults in the current study). Similarly, prior studies on opioid abuse and misuse have consistently identified a significant relationship between physical abuse [18,36] but less so for sexual or emotional abuse [37,38]. Overall, this emerging body of research complements earlier research on personal characteristics as correlates of opioid use and opioid prescription use by providing a more systematic explanation of the processes that drive opioid use. These findings also highlight the need for additional research on how often and extended exposure to childhood abuse and the consideration of different types that would influence opioid prescription use.

Furthermore, our findings that only Whites with a history of childhood physical abuse are at a higher risk of opioid prescription usage align with the documented disparities in access to prescription opioids for Blacks and Whites with comparable pain conditions [25,27]. This discrepancy may inadvertently contribute to adverse consequences, including the perpetuation of the misconception that Black individuals are shielded from the opioid crisis and the potential for severe undertreatment or improper management of medical conditions for Blacks, even when they are in dire need [39]. Our study underscores the pressing necessity to scrutinize race-specific pathways linking ACEs to opioid prescription usage patterns.

The current study has certain limitations. First, the sample of opioid prescription users was relatively small; thus, the results of interaction between childhood abuse experiences and race warrant cautious interpretation and generalization. Second, the assessment of childhood abuse was both retrospective and self-reported, and participants might have reported inaccurate information. Although the Childhood Trauma Questionnaire is a well-established and reliable measure of childhood adversity, retrospective self-reported data may still be vulnerable to biases, such as those stemming from motivation or memory distortions [40]. Third, our dataset does not have information on non-prescription opioid use. There could be individuals who only use illicit/recreational opioids such as heroin, and if they are more likely from certain racial and ethnic groups, our non-inclusion of these variables may have influenced our findings. In addition, due to the dearth of studies on opioid prescription use and childhood abuse, our interpretation of the findings is primarily guided by the literature on opioid misuse and abuse. Future research could also include more details on opioid prescription use (e.g., dosage and duration) to create a more comprehensive picture of the association between childhood abuse and the use of opioid prescriptions. In addition, due to the small sample size of minority racial/ethnic groups in the MIDUS national sample (e.g., Hispanic, Pacific Islanders, Native Americans, Asians), the current study included only non-Hispanic Whites and non-Hispanic Blacks. Future studies with greater representation of racial and ethnic minority groups could enhance the understating of subgroup differences across various racial and ethnic groups. Lastly, the main scope of this study was to understand whether childhood abuse experience serves as an important early life event shaping the likelihood of opioid prescription use in adulthood. Therefore, although guided by the life course perspective, we controlled only for a few primary factors, such as education and health status, which may lie in the sequential pathway between childhood abuse and opioid prescription use. Also, due to limited information in secondary data, the current study examined opioid prescription use at a singular time point rather than long-term use and its association with adverse childhood experiences. Future studies that examine possible mechanisms through which negative childhood experiences are associated with opioid prescription use in adulthood (e.g., work, marriage, health behaviors, mental and physical health) will improve our understanding of the subject area.

The present study also has important strengths. First, by utilizing a national sample of adults ages 35–84 who use prescription medications, our findings are generalizable to prescription medication users across a wide age range and extend prior studies that focused on young adults and clinical samples. Second, participants were instructed to provide all medication materials during the data collection, and the study staff used these materials to record a comprehensive list of participants’ medications. Thus, the medication information is far more reliable than self-reported measures, moving the work beyond self-report. In addition, we used three different types of childhood abuse measured with five items each, which also included information on the severity. This approach offers an improvement over prior research that used a single binary indicator of childhood abuse history, as it enhances both the reliability and the level of detail of the data.

The current study results have important implications for mental health practices and policies. There has been growing concern that the use of opioid prescription medications is a strong risk factor for opioid addiction, abuse, or misuse in the future [4,20]. About 21–29% of people prescribed opioids for chronic pain reported misusing them, with roughly 8–12% developing an opioid use disorder [41]. Mounting evidence of the strong association between childhood abuse and opioid use disorder in adulthood has driven recent efforts to develop interventions targeting people who experienced childhood abuse [42]. In this sense, trauma-informed therapy may be necessary for treatment programs to prevent opioid addiction among opioid prescription users who have experienced childhood abuse, particularly physical abuse [43]. As suggested in the CDC opioid prescription guidelines for patients with chronic pain [44], increased monitoring and cautious dose titration can be implemented for people with previous physical childhood abuse experiences. Efforts also include careful monitoring of the history of childhood abuse and referral for its recovery in adulthood. Clinical settings should be developed to allow people to comfortably discuss their past experiences when needed without the fear of differential treatment. To deliver more comprehensive services, child protective services could identify young individuals at high risk of opioid prescription use and offer this group education on opioid misuse/abuse prevention and strategies for coping with childhood abuse. Further, programs directed at health professionals in the field, including physicians, psychologists, health navigators, and social workers, should seek to enhance their awareness of and knowledge about opioid prescription use and the possible risk of opioid misuse/abuse. Finally, health professionals and service providers should be aware of the adverse effects of childhood abuse, particularly on opioid prescription use, so that they can provide optimal support for individuals at greater risk of experiencing opioid prescription-related problems.

## Supporting information

S1 File The supplementary document presents the Childhood Trauma Questionnaire (CTQ). (DOCX)
