# Supplementary material for: Childhood abuse and opioid prescription use in adulthood: Differences between non-Hispanic Whites and non-Hispanic Blacks in the United States
Source: PLoS One. 2023 Sep 21;18(9):e0291752. doi: 10.1371/journal.pone.0291752 (PMC10513245; doi:10.1371/journal.pone.0291752)
Supplement: S1 File — (DOCX) [file pone.0291752.s001.docx]

CHILDHOOD TRAUMA QUESTIONNAIRE (CTQ)

Emotional Abuse (5 items)

People in my family called me things like “stupid,” “lazy,” or “ugly.”

I thought that my parents wished I had never been born.

People in my family said hurtful or insulting things to me.

I felt that someone in my family hated me.

I believe that I was emotionally abused.

Physical Abuse (5 items)

I got hit so hard by someone in my family that I had to see a doctor or go to the hospital.

People in my family hit me so hard that it left me with bruises or marks.

I was punished with a belt, a board, a cord, or some other hard object.

I believe that I was physically abused.

I got hit or beaten so badly that it was noticed by someone like a teacher, neighbor, or doctor.

Sexual Abuse (5 items)

Someone tried to touch me in a sexual way, or tried to make me touch them.

Someone threatened to hurt me or tell lies about me unless I did something sexual with them.

Someone tried to make me do sexual things or watch sexual things.

Someone molested me.

I believe that I was sexually abused.

(1 = Never true, 2 = Rarely true, 3 = Sometimes true, 4 = Often true, 5 = Very often true).
